# Assessing likelihood of product use for snus with modified-risk information among adult current cigarette smokers, former tobacco users, and never tobacco users

**DOI:** 10.1016/j.abrep.2019.100208

**Published:** 2019-07-26

**Authors:** Karen Gerlach, Saul Shiffman, Deena Battista, Michael Polster, Geoffrey Curtin

**Affiliations:** a PinneyAssociates, Inc., 201 North Craig Street, Suite 320, Pittsburgh, PA 15213, United States of America; b NAXION, 1835 Market Street, Philadelphia, PA 19103, United States of America; c RAI Services Company, 401 North Main Street, Winston-Salem, NC 27101, United States of America

**Keywords:** Likelihood of use, Modified risk, Snus, Tobacco harm reduction

## Abstract

**Introduction:**

Switching from cigarettes to snus by smokers unlikely to quit would be expected to benefit overall population health, with any potential benefit needing to be weighed against potential harms from snus use by tobacco non-users and smokers likely to quit. This study evaluates likelihood of snus use among tobacco users and non-users provided modified-risk information.

**Methods:**

An online sample of 11,302 U.S. adults was randomized to view advertisements for snus that either provided modified-risk information or only described snus. Intent to purchase ratings were converted to projected purchase (use) rates using an empirically derived algorithm.

**Results:**

Projected product use for snus was significantly higher among current smokers than former or never tobacco users (*p* < 0.0001) for both the modified-risk and control information. A significant interaction effect between information and tobacco user group (*p* < 0.0001) indicated the modified-risk information differentially increased projected use among smokers (8.2% vs. 6.9%), with much lower projections for both the test and control information among former (1.2%) and never tobacco users (0.4%). Among never users, projected use was highest among those susceptible to smoking. These findings were generally similar for young adults, ages 18–24. Smokers expecting to quit who viewed modified-risk information had lower projected use (4.2%) than those not expecting to quit (8.7%).

**Conclusions:**

Results suggest that providing modified-risk information for snus is unlikely to increase use among those not using tobacco. Interest in snus was greatest among current smokers who would benefit by switching to snus as communicated in the modified-risk advertisement.

## Highlights

• We tested the effect of communicating the reduced risk of snus compared to smoking.
• Such communication increased likely use of snus among smokers.
• Likely use of snus did not increase in never and former tobacco users.
• Projected use of snus was highest among smokers not likely to quit.
• Providing risk information is unlikely to cause harm and may confer benefits.

## 1 Introduction

Tobacco products range in health risk, with cigarettes presenting the greatest risk and non-combustible products presenting comparably less risk (Zeller, 2013). Snus, a smokeless tobacco product with low levels of tobacco-specific nitrosamines, has been available in Sweden for decades. Many Swedish smokers (particularly males) have switched completely from cigarettes to snus, resulting in lower rates of tobacco-related diseases, including lung cancer rates that are the lowest in Europe (Foulds, Ramstrom, Burke, & Fagerstrom, 2003; Rodu & Cole, 2009). Levy and colleagues estimated that snus presents about 10% of the mortality risk of smoking (Levy, Mumford, Cummings, et al., 2004). Encouraging smokers to switch to snus would benefit both individual and population health, but may require educating smokers about the potential to reduce their risk by switching.

Smokers hold misperceptions about the relative risks of smokeless tobacco versus cigarettes, with most studies reporting that few U.S. smokers believe smokeless tobacco (Borland, Cooper, McNeill, O'Connor, & Cummings, 2011; Czoli, Fong, Mays, & Hammond, 2017) and snus (Fong, Elton-Marshall, Driezen, et al., 2016; Kaufman, Mays, Koblitz, & Portnoy, 2014; Regan, Dube, & Arrazola, 2012) are less harmful than smoking. Smokers' risk perceptions are more accurate after trying smokeless tobacco (Hatsukami, Vogel, Severson, Jensen, & O'Connor, 2016), and providing them with relative-risk information can reduce misperceptions and increase interest in trying such products (Borland, Li, Cummings, et al., 2012).

Carpenter et al. provided snus free to smokers not interested in quitting, along with information on why it is less harmful than cigarettes, and found that frequent users of snus were more likely to try to quit smoking and succeed than those who did not use snus (Carpenter, Wahlquist, Burris, et al., 2017). Another study found snus was about as effective as nicotine gum for achieving 7-day smoking abstinence in smokers interested in switching to snus to reduce harm (Hatsukami, Severson, Anderson, et al., 2016).

In the U.S., tobacco products can be advertised with modified-risk claims, provided the available evidence demonstrates that the product would “[b]enefit the health of the population as a whole taking into account both users of tobacco products and persons who do not currently use tobacco products” (Family Smoking Prevention and Tobacco Control Act, 2009). A population health benefit would be expected if smokers not likely to quit instead switched completely to a modified-risk product, although use by non-tobacco users or smokers who would have otherwise quit could adversely affect population health. Assessing the effects of communicating modified-risk information for a tobacco product cannot be fully determined in advance but can be assessed in terms of the likelihood of product use by tobacco users and non-users.

This study examined likelihood of use for a snus product with modified-risk information among cigarette smokers and tobacco non-users (former and never users).

## 2 Methods

### 2.1 Design

Participants were randomized to view either a snus advertisement with modified-risk information (test condition) or an advertisement that described the product but did not include information on comparative risk (control condition). Respondents then rated their likelihood to purchase the product for trial, and those intent ratings, along with projected purchase rates, were compared among tobacco user groups. This study was not reviewed by an Institutional Review Board since, in accordance with the Code of Federal Regulations 45 Part 46.101.b, survey research that is anonymous or does not solicit subject-identified sensitive information that could harm participants is considered exempt (U.S. Department of Health and Human Services, 2017).

### 2.2 Sample

Data were collected in August and September 2015 from 11,302 U.S. adults sampled from the Research Now national online panel of approximately three million individuals who volunteer for surveys (Research Now, 2017). Quota sampling was undertaken to ensure representation across key demographics (i.e., gender, age, race/ethnicity, education, and geographic region), and the test and control samples were separately weighted on those demographic factors to match the U.S. population (U.S. Census Bureau, n.d.; U.S. Census Bureau, 2014).

To mask the study's focus, screening questions about tobacco use were embedded with questions about regular use of beer or malt-based beverages, bottled water, and nutritional supplements/vitamins. Panelists responding to the invitation were assessed for demographic characteristics, tobacco use histories, and expectations to quit tobacco use (among current users). Respondents ages 18–75, legally eligible to purchase tobacco in their state, and not current users of the Camel Snus brand in the advertisement, were eligible to participate. Quota sampling was used to fill three groups based on self-defined tobacco use, i.e., current regular tobacco users (*n* = 3466), former regular tobacco users (*n* = 3379), and never regular tobacco users (*n* = 4457); tobacco user groups were balanced between test and control conditions. “Potential quitters” (*n* = 497) were current regular tobacco users who stated they expected not to be using any tobacco nine months after the survey, matching the interval used in the empirically derived algorithm for projecting purchase rates based on intent ratings (Algorithm Description in Supplemental Material).

### 2.3 Message exposure

Individuals viewed either the test or control advertisement for Camel Snus comprised of three color images, one above the other on-screen. The bottom fifth of each advertisement included one of four randomly rotated government-mandated warnings for smokeless tobacco. After a minimum viewing time of 10 s, respondents advanced to questions presented on separate screens; respondents could not go back to review the advertisement.

The test advertisement included information on the benefits for smokers switching completely from cigarettes to snus (i.e., reduced risk of lung cancer, respiratory disease, heart disease, and oral cancer). In addition to this modified-risk information, the test advertisement included balancing information intended to communicate, for example, that less risk does not mean no risk and no tobacco product is safe. The control advertisement did not include any modified-risk information or balancing information (Table 1; Advertisements in Supplemental Material).

**Table 1:** Comparison of information presented in test and control conditions (advertisements).

Information	Test condition	Control condition
Camel Snus Information		
What is Camel Snus? Camel Snus (rhymes with “moose”) is finely ground premium tobacco in a soft fleece pouch.	X	X
How is it different? Many smokeless tobacco products, like dip and chew, are fermented loose tobacco. Sure, they're smoke-free, but they can get messy and require spitting. Snus is different. It's smoke-free, mess-free and spit-free. Camel Snus is heat-treated, not fermented, and crafted with four main ingredients: tobacco, water, salt and flavoring.	X	X
How do I use it? Slide a pouch under your upper lip.	X	X
Taste the real, premium tobacco.	X	X
Dispose of the pouch in the trash when you are finished.	X	X
I'm a smoker. Why should I switch? Switching to snus means …		
No lingering smoke smell Hassle-free tobacco	X	X

Mandated Warnings		
1 of 4 government-mandated warning statements for smokeless tobacco products (rotated) • WARNING: This product can cause mouth cancer. • WARNING: This product can cause gum disease and tooth loss. • WARNING: This product is not a safe alternative to cigarettes. • WARNING: Smokeless tobacco is addictive.	X	X

Modified-Risk Information		
Switch completely from cigarettes to Camel Snus.	X	
No smoke = less risk	X	
Smokers who SWITCH COMPLETELY from cigarettes to Camel Snus can greatly reduce their risk of lung cancer, oral cancer, respiratory disease, and heart disease.	X	
Scientific studies have shown that Camel Snus contains less of the harmful chemicals than cigarette smoke.	X	
Camel Snus is smoke-free, so there are no second-hand smoke risks for those around you.	X	
I'm a smoker. Why should I switch? Switching to snus means …		
Less of the harmful chemicals found in cigarette smoke Less risk for you and those around you	X	
But if you're not going to quit using tobacco products, you should think about switching to Camel Snus.	X	

Balancing Information		
No tobacco product is safe.	X	
Like all tobacco products, Camel Snus contains nicotine and is addictive.	X	
Adults who do not use or have quit using tobacco products should not start.	X	
Minors and pregnant women should never use tobacco products.	X	
If you're a smoker concerned about the health risks from smoking, the best choice is to quit. A good place to begin is talking with a healthcare provider.	X	

### 2.4 Assessment

After viewing either the test or control advertisement, respondents rated their intent to purchase snus (1–10 scale; 1=“definitely would not purchase to try it” and 10=“definitely would purchase to try it”). Current smokers not expecting to quit and expressing *any* interest in snus (intent rating > 1) were asked how they intended to use snus (instead of current tobacco product, in place of some of current tobacco product, in addition to current tobacco product, or don't know). Potential quitters expressing any interest in snus were asked their reason for interest in snus (to help them quit, to use in situations where their current tobacco product cannot be used, just curious about it, or don't know).

Never tobacco users answered questions about susceptibility to smoking. Those not responding “definitely no” to the questions, “Do you think you will smoke a cigarette in the next year?” and “If one of your best friends were to offer you a cigarette, would you smoke it?” were considered “susceptible” to smoking cigarettes (Pierce, Choi, Gilpin, Farkas, & Merritt, 1996).

### 2.5 Analysis

Primary outcomes for this study were (1) intent to purchase ratings and (2) projected purchase rates. An empirically derived algorithm was used to convert intent ratings (1–10 scale) into projected purchase rates (a proxy for product use). This algorithm was based on a logistic regression model that used respondents' tobacco use status, gender, and age as moderators (Algorithm Description in Supplemental Material).

The primary objective was to estimate the likelihood of use (based on projected product use) for snus after viewing the modified-risk information (test) compared to the control condition by tobacco use status, i.e., current smokers (currently use cigarettes, roll-your-own cigarettes, or tobacco heating cigarettes on a “regular basis”), and former and never tobacco users. Factorial analysis of variance was used to test differences in mean intent ratings and projected product use among tobacco user groups, and by test versus control condition. A main effect for tobacco user group reflects differences in projected product use across tobacco user groups (across test and control conditions). A main effect for the information reflects differences in projected product use for the test versus control condition (across tobacco groups). The interaction effect reflects differences for the test versus control condition across different tobacco user groups.

Additional analyses were done among potential quitters, never tobacco users susceptible to smoking, and young adults ages 18–24, who are of particular interest as their tobacco use behavior might be more amenable to change.

## 3 Results

### 3.1 Demographics

Demographic characteristics were nearly identical between the test (*n* = 5647) and control (*n* = 5655) conditions, with no significant differences. The weighted samples each mirror the U.S. population for gender (female, 51%), age (18–24: 12%; 25–44: 37% test/36% control; 45–64: 36% test/37% control; and, 65–75: 15%), and race/ethnicity (non-Hispanic White: 64%; non-Hispanic Black: 12%; Hispanic: 16%; and, non-Hispanic Asian/Other/Multi-race: 8%). Less than half (41%) had a high school education or less, and 30% had a college degree.

### 3.2 Tobacco use

Test and control conditions were balanced on tobacco use status, potential quitting (among smokers), and susceptibility to smoking (among never tobacco users). The majority (~84%) of current tobacco users were smokers, with approximately 80% reporting daily smoking. Approximately 11% used smokeless tobacco (about 60% non-daily), with about half reporting use of a snus brand other than Camel Snus.

### 3.3 Intent to purchase ratings and projected product use

#### 3.3.1 Current smokers, and former and never tobacco users

Intent to purchase ratings and projected product use were very low among former and never tobacco users, and were comparatively higher among current smokers. There was a significant (*p* < 0.05) interaction effect between condition and tobacco user group for mean intent to purchase ratings. The modified-risk information differentially increased intent to purchase among current smokers compared to former and never tobacco users (Table 2). There were also significant main effects for condition (*p* < 0.05) and tobacco user group (*p* < 0.0001). Intent to purchase snus was significantly greater (*p* < 0.0001) among current smokers than among either former or never tobacco users.

**Table 2:** Intent to purchase ratings and projected product use among current smokers, and former and never tobacco users.

	Intent to purchase ratings (1–10 rating scale) Mean (95% CI)				Projected product use % (95% CI)			
	Test condition	Control condition	Overall	**C** = Main effect of condition **G** = Main effect of tobacco user group **I**=Interaction effect	Test condition	Control condition	Overall	**C** = Main effect of condition **G** = Main effect of tobacco user group **I**=Interaction effect
Tobacco use status								
Current smokers	3.7 (3.6–3.9) (*n* = 1333)	3.4 (3.2–3.5) (*n* = 1328)	3.5 (3.4–3.7)	**C:***p* = 0.0151 **G:***p* < 0.0001 **I:***p* = 0.0313	8.2% (6.0–10.9%) (*n* = 1333)	6.9% (5.1–9.4%) (*n* = 1328)	7.5% (5.6–10.2%)	**C:***p* < 00.0001 **G:***p* < 0.0001 **I:***p* < .0001
Former tobacco users	1.6 (1.4–1.7) (n = 1689)	1.6 (1.4–1.7) (*n* = 1690)	1.6 (1.5–1.7)		1.2% (0.6–2.4%) (*n* = 1689)	1.2% (0.6–2.4%) (*n* = 1690)	1.2% (0.6–2.4%)	
Never tobacco users	1.7 (1.6–1.7) (n = 2225)	1.7 (1.6–1.7) (n = 2232)	1.7 (1.6–1.7)		0.4% (0.2–0.7%) (*n* = 2225)	0.4% (0.2–0.7%) (*n* = 2232)	0.4% (0.2–0.7%)	
Overall	2.3 (2.3–2.4)	2.2 (2.1–2.3)			1.5% (1.0–2.3%)	1.4% (0.9–2.1%)		

Expected quitting among current smokers								
Potential quitters	2.5 (2.0–2.9) (*n* = 184)	2.3 (1.7–2.8) (*n* = 155)	2.4 (2.0–2.7)	**G:** p < 0.0001	4.2% (2.9–6.1%) (n = 184)	4.0% (2.7–5.8%) (n = 155)	4.1% (2.8–5.9%)	**G:** p < 0.0001
Not potential quitters	3.9 (3.7–4.1) (n = 1149)	3.5 (3.3–3.7) (*n* = 1173)	3.7 (3.6–3.8)		8.7% (6.5–11.6%) (*n* = 1149)	7.3% (5.4–9.8%) (*n* = 1173)	8.0% (5.9–10.7%)	
Overall	3.2 (2.9–3.4)	2.9 (2.6–3.2)			8.2 (6.0–10.9)	6.9 (5.1–9.4)		

Susceptibility to smoking among never tobacco users								
Susceptible to Smoking	3.3 (3.1–3.4) (*n* = 421)	3.1 (2.9–3.2) (*n* = 439)	3.2 (3.1–3.3)	**G:** p < 0.0001	0.8% (0.5–1.5%) (n = 421)	0.7% (0.4–1.3%) (n = 439)	0.8% (0.4–1.4%)	**C:***p* = 0.0141 **G:***p* < 0.0001 **I:***p* = 0.0083
Not susceptible to smoking	1.3 (1.2–1.3) (n = 1804)	1.3 (1.2–1.4) (*n* = 1793)	1.3 (1.2–1.3)		0.3% (0.1–0.5%) (*n* = 1804)	0.3% (0.2–0.5%) (*n* = 1793)	0.3% (0.1–0.5%)	
Overall	2.3 (2.2–2.4)	2.2 (2.1–2.3)			0.4% (0.2–0.7%)	0.4% (0.2–0.7%)		

There was also a significant (p < 0.0001) interaction effect between condition and tobacco user group for projected product use, indicating the modified-risk information differentially increased projected use among current smokers. There were significant main effects for condition (*p* < 0.0001) and tobacco user group (p < 0.0001). Projected product use was significantly greater (*p* < 0.0001) among current smokers than either former or never tobacco users; and, former tobacco users had significantly greater (p < 0.0001) projected use than never tobacco users.

#### 3.3.2 Potential quitters

Current smokers who expected to quit had lower intent to purchase ratings and projected product use than smokers not expecting to quit. There was no significant interaction effect between condition and quit expectations among current smokers for either intent to purchase or projected product use; that is, the modified-risk information did not differentially impact projected use among potential quitters (Table 2). There was no main effect for condition when examining either of the primary outcomes, but there were main effects for tobacco user group. Potential quitters had significantly less interest in purchasing snus (*p* < 0.0001) and lower projected product use (p < 0.0001) than smokers who did not expect to quit.

#### 3.3.3 Never tobacco users susceptible to smoking

Among never tobacco users, both intent to purchase and projected product use were low. There was no significant interaction effect between condition and susceptibility to smoking for intent to purchase, nor was there a main effect for condition (Table 2). There was, however, a main effect for susceptibility to smoking, with those susceptible rating their intent to purchase snus significantly higher (*p* < 0.0001) than those not susceptible.

For projected product use, there was a significant (*p* < 0.01) interaction effect between condition and susceptibility to smoking, in addition to both a main effect for condition (*p* < 0.05) and tobacco user group (*p* < 0.0001). That is, the modified-risk information differentially increased projected product use among the never tobacco users susceptible to smoking, but not among those not susceptible to smoking.

#### 3.3.4 Young adults

Young adult current smokers had higher intent to purchase ratings and projected product use than both young adult former and never tobacco users. Among young adults there was no significant interaction effect between condition and tobacco user group for intent to purchase (Table 3), indicating the modified-risk information did not differentially impact purchase intent among young adult never tobacco users. There was no main effect for condition, but there was a significant (*p* < 0.0001) main effect for tobacco user group. Intent to purchase ratings were significantly higher (p < 0.0001) among young adult current smokers than former and never tobacco users; and, significantly higher (*p* < 0.05) among former compared to never users.

**Table 3:** Intent to purchase ratings and projected product use among young adult (ages 18–24) current smokers, and former and never tobacco users.

	Intent to purchase ratings (1–10 rating scale) Mean (95% CI)				Projected product use % (95% CI)			
	Test condition	Control condition	Overall	**C** = Main effect of condition **G** = Main effect of tobacco user group **I**=Interaction effect	Test condition	Control condition	Overall	**C** = Main effect of condition **G** = Main effect of tobacco user group **I**=Interaction effect
Tobacco use status								
Current smokers	5.2 (4.5–5.9) (*n* = 68)	4.1 (3.3–4.8) (*n* = 64)	4.6 (4.1–5.1)	**G:** p < 0.0001	14.6% (10.6–19.7%) (*n* = 68)	11.1% (8.0–15.3%) (n = 64)	12.9% (9.3–17.5%)	**C:***p* = 0.0397 **G:** p < 0.0001 **I:***p* = 0.0002
Former tobacco users	2.4 (1.4–3.4) (*n* = 38)	2.9 (1.9–4.0) (n = 34)	2.7 (1.9–3.4)		2.6% (1.0–6.4%) (n = 38)	3.1% (1.2–7.7%) (n = 34)	2.9% (1.1–7.0%)	
Never tobacco users	1.8 (1.6–2.0) (*n* = 331)	1.8 (1.6–2.1) (*n* = 344)	1.8 (1.7–2.0)		0.5% (0.3–0.8%) (n = 331)	0.4% (0.2–0.7%) (n = 344)	0.4% (0.2–0.8%)	
Overall	3.1 (2.7–3.6)	2.9 (2.5–3.4)			1.9% (1.3–2.9%)	1.5% (1.0–2.3%)		

Susceptibility to smoking among never tobacco users								
Susceptible to smoking	3.6 (3.2–4.0) (*n* = 87)	3.4 (3.0–3.8) (*n* = 88)	3.5 (3.2–3.8)	**G:** p < 0.0001	1.0% (0.6–1.8%) (*n* = 87)	0.8% (0.5–1.4%) (n = 88)	0.9% (0.5–1.6%)	**G:***p* < 0.0001 **I:***p* = 0.0258
Not susceptible to smoking	1.2 (0.9–1.4) (*n* = 244)	1.3 (1.1–1.5) (*n* = 256)	1.2 (1.1–1.4)		0.2% (0.1–0.5%) (n = 244)	0.3% (0.1–0.5%) (n = 256)	0.3% (0.1–0.5%)	
Overall	2.4 (2.2–2.6)	2.3 (2.1–2.6)			0.5% (0.3–0.8%)	0.4% (0.2–0.7%)		

Among young adults, only 14 (6 test condition; 8 control condition) were assessed to be potential quitters, precluding analysis within this age group by potential quitter status.

There was, in contrast, a significant (*p* < 0.001) interaction effect between condition and tobacco user group for projected product use. The modified-risk information differentially increased projected product use among young adult current smokers compared to former and never tobacco users. There was also a significant main effect for condition (*p* < 0.05) and tobacco user group (*p* < 0.0001). Thus, young adult current smokers had significantly higher projected product use than both former and never tobacco users, and former users had significantly higher projected product use than never users (all *p* < 0.0001).

Among young adult never tobacco users, there was no significant interaction effect between condition and susceptibility to smoking for intent to purchase; the modified-risk information did not differentially impact intent to purchase among young adults not susceptible to smoking (Table 3). There was no main effect for condition, but there was a significant (*p* < 0.0001) main effect for susceptibility to smoking; susceptible never tobacco users were significantly more interested in using snus than those not susceptible to smoking.

There was a significant (*p* < 0.05) interaction effect between condition and susceptibility to smoking for projected product use among young adults. The modified-risk information differentially increased projected product use among young adults who were susceptible to smoking compared to those not susceptible. There was no main effect of condition, but there was a significant (*p* < 0.0001) main effect by susceptibility; susceptible never tobacco users had significantly higher projected product use than those not susceptible to smoking.

### 3.4 Anticipated use behavior among smokers not expecting to quit

Smokers not expecting to quit expressing any interest in snus (intent rating > 1; test: *n* = 604; control: *n* = 583) were asked how they would anticipate using snus. The test and control groups did not differ significantly in their anticipated use behaviors, with about half expecting to replace all (test: 22%, 17.5%–26.2%; control: 20%, 16.1%–24.6%) or some (test: 30%, 25.2%–33.9%; control: 26%, 22.0%–30.4%) of their cigarette use with snus. Similar proportions in the test and control groups expected to add snus to their current tobacco use (test: 22%, 17.8%–26.4%; control: 27%, 22.9%–32.0%).

### 3.5 Reasons for potential quitters' interest in using snus

To understand why some potential quitters had any interest in using snus (intent rating > 1; test: *n* = 62; control: *n* = 47), they were asked their reason for wanting to use snus. For the test condition, 51% (36.1%–66.6%) stated their reason was “to help me quit,” compared to 34% (17.5%–51.3%) in the control condition. Reasons cited more often in the control versus test condition were curiosity (46% versus 28%) and to use in situations where their current product could not be used (12% versus 5%). However, these differences between conditions were not significant.

## 4 Discussion

The population health benefit expected from use of a tobacco product that presents less risk than cigarettes depends on who uses the product. Use of a product such as snus instead of cigarettes by current smokers not likely to quit would benefit both their individual health and population health. Conversely, use by tobacco non-users or current smokers likely to quit could be harmful (Levy et al., 2004; Zeller, 2013). The current study was conducted to assess the likelihood of product use for snus with modified-risk information among these populations.

Overall, the results indicated that interest in snus use was low, even among current smokers, and was not increased by exposure to the modified-risk information. Respondents' intent to purchase ratings and projected product use indicated that the likelihood of use for snus was greatest among smokers not likely to quit – who could benefit from switching – and was very low among former and never tobacco users. Projected product use among smokers who viewed the modified-risk information (8.2%) was 20 times higher than among never tobacco users (0.4%), and almost seven times higher than among former tobacco users (1.2%). Moreover, the modified-risk information differentially increased intent to purchase and projected product use among current smokers, compared to former and never tobacco users, and did not significantly or differentially increase projected use among the populations whose risk could be increased by using snus (non-users and smokers likely to quit).

Slightly more than half of current smokers expecting to quit who viewed the modified-risk information and expressed any interest in snus indicated their motive for using snus was “to help me quit,” suggesting they may view snus not as an alternative to quitting but as a possible aid in quitting. Among current smokers who were not expecting to quit and who expressed any interest in using snus, one in five indicated they intended to replace all of their cigarettes with snus. About one-third indicated they would replace some of their cigarettes with snus; reducing smoking may have health benefits (Krautter, Chen, & Borgerding, 2015), but this is less clear. Finally, about one in five indicated they would use snus in addition to their current cigarettes; this unintended use behavior could potentially confer some benefit if it leads to a decline in total cigarette consumption and eventually smoking cessation (Frost-Pineda, Appleton, Fisher, Fox, & Gaworski, 2010). Communicating the importance of complete switching is necessary, and post-marketing surveillance would be needed to monitor the behavior of smokers who adopt any modified-risk tobacco product.

We examined responses among young adults, whose tobacco use patterns may not yet be fully established and whose behavior might then be more influenced by modified-risk information about tobacco. Young adult current smokers showed the highest level of interest in using snus. This may have a beneficial population health effect, in that smokers gain the greatest benefit from quitting smoking as early as possible (Jha, Ramasundarahettige, Landsman, et al., 2013). Conversely, concerns have been raised about the adoption of smokeless tobacco by non-tobacco users serving as a gateway to smoking (Joffer et al., 2014). However, population studies do not consistently find such an effect, with several studies suggesting that adoption of smokeless tobacco is associated with a lower risk of subsequent smoking (Lund, Scheffels, & McNeill, 2011; Ramstrom, Borland, & Wikmans, 2016). Interest in snus among young adult never tobacco users was very low (projected use of 0.4%), and was not increased by providing modified-risk information (projected use of 0.5%).

The findings suggest that there is low interest in using snus, even among current smokers, consistent with data on use of snus in the U.S. (King, Dube, & Tynan, 2012) Motivating smokers to switch completely to snus may depend on persuading them that snus presents less risk than cigarettes. Both smokers and non-smokers have misperceptions about the absolute and relative risks of smokeless tobacco, with many believing it is at least as hazardous as smoking (Fong et al., 2016; Kaufman et al., 2014; Kiviniemi & Kozlowski, 2015; Regan et al., 2012). Multiple exposures and consistent information from multiple sources, including sources more credible than a tobacco advertisement, may be needed for smokers to appreciate the potential for products such as snus to reduce health risks (Byrne, Guillory, Mathios, Avery, & Hart, 2012; Harris Interactive, 2013). Similarly, exposure to multiple messages about the importance of switching completely from cigarettes to snus may be necessary to promote such behavior.

This study had some limitations. The sample was drawn from an online panel, and may not be fully representative of the U.S. population. However, a majority of U.S. consumers are online (Perrin & Duggan, 2015), and online panels can produce reasonable population estimates (Farrell, 2010). Moreover, the sample was recruited and weighted to represent the demographics of the U.S. population. The study was conducted among adults who could legally purchase tobacco, and did not assess those under the legal age to purchase tobacco. However, data among young adults found little interest in snus among those not using tobacco, and modified-risk information did not increase their interest.

Respondents were shown the information online; online exposure is often used to evaluate communications (Sullivan & O'Donoghue, 2015), and there is little reason to think the current findings are not generalizable to other media. Importantly, the study measured the effects of one exposure to modified-risk information during the course of a survey, as opposed to the effects of multiple exposures over time in a real-world advertising context.

The study assessed self-reported interest in purchasing snus, and did not assess actual use. An empirically derived algorithm was used to project purchase (use) rates based on rated intent to purchase, but that algorithm was based on and validated within the context of cigarettes, not a smokeless tobacco product. Subsequent studies suggested that actual purchase rates are over-projected by the algorithm, though the differential rates among the different tobacco user groups is accurately captured (Algorithm Description in Supplemental Material). The algorithm also projects initial purchase, not long-term persistence, which is important for the expected population health benefit of switching to snus. Carpenter and colleagues found that, among smokers who tried snus, 8% reported any use after 6-months and only 4% after 12-months (Carpenter et al., 2017). Thus, projected purchase rates over-estimate persistent use.

This study also had considerable strengths. The sample was large, diverse, and sampled and weighted to match the demographic characteristics of U.S. adults. Intent to purchase ratings were translated into projected purchase rates, taking into account empirical observations about how intent ratings translate into real-world behavior (via the algorithm). The observed differences in projected product use between current smokers and non-tobacco users were large and statistically reliable. Additional testing of two different variations of the modified-risk information provided similar results (U.S. Food and Drug Administration, n.d.), suggesting the results are robust.

Smokers not likely to quit were the group most interested in snus, albeit at low rates. Smokers may need more than one exposure to the modified-risk information to appreciate the potential of completely switching to snus to reduce health risks, which would benefit individual and overall population health. Importantly, the finding that modified-risk information did not increase interest in snus among those who would be harmed by taking up snus (never and former tobacco users, and smokers likely to quit) suggests that providing modified-risk information is unlikely to cause harm.

## Funding

This work was supported by RAI Services Company (RAIS).

## Role of funding sources

Funding for this study was provided by RAI Services Company. The study was conducted for a regulatory submission to the U.S. Food and Drug Administration; as such, the study sponsor has a contributing role in the study design, interpretation of the data, writing the manuscript, and decision to submit the paper for publication. The analysis was conducted by Pinney Associates, which had complete access to the data.

## Contributors

Drs. Polster and Curtin designed the study; Dr. Polster was responsible for data collection; Drs. Polster, Battista and Shiffman had contributing roles during the analysis of data; all authors had a role in interpreting the data, writing the manuscript, and approving the paper for publication.

## Declaration of Competing Interest

Dr. Curtin is employed by RAIS, a wholly owned subsidiary of Reynolds American Inc., acquired by British American Tobacco in July 2017, and whose operating companies market smokeless tobacco products. Through PinneyAssociates, Drs. Shiffman, Gerlach and Battista provide consulting services on smoking cessation and tobacco harm minimization (including nicotine replacement therapy and electronic vapor products, but not combustible cigarettes) to RAIS. Dr. Shiffman also owns an interest in intellectual property for a novel nicotine medication that has neither been developed nor commercialized. Dr. Polster is employed by NAXION, which provides consulting services to RAIS.

## Appendix A Supplementary data

Supplementary material Image 1
