# Supplementary material for: Assessing likelihood of product use for snus with modified-risk information among adult current cigarette smokers, former tobacco users, and never tobacco users
Source: Addict Behav Rep. 2019 Jul 26;10:100208. doi: 10.1016/j.abrep.2019.100208 (PMC6710553; doi:10.1016/j.abrep.2019.100208)
Supplement: Supplementary file 1 — Supplementary material [file mmc1.docx]

**Supplemental Material**

**Algorithm Description**

Rated intent to use is often used as a proxy for subsequent use in consumer studies. However, stated *intent* to purchase does not always translate into *actual* purchase. Accordingly, a predictive algorithm was developed to transform the ‘intent to purchase’ ratings into projected purchase rates for the tobacco product. This algorithm was developed empirically by comparing *pre*-launch survey ratings of intent to purchase a tobacco product (a new brand of cigarettes) with actual (self-reported) subsequent purchase of that product *post*-launch. A total of 15,393 consumers (roughly evenly distributed among current regular, former regular, and never regular tobacco users) from an online web-panel participated in a 5-minute survey in which they were presented an image of a new cigarette and asked how likely they would be to purchase the product, rating intent to purchase on a 10-point scale. Nine months later (to allow for distribution and uptake of the product), all respondents were invited to participate in another 5-minute survey about which cigarettes, if any, they had purchased in the past nine months. A total of 8,328 consumers participated in the second (follow-up) survey and constitute the sample for development of the algorithm.

The projective algorithm was created using a survey-weighted multivariable logistic regression model that used initial ratings, demographic variables (i.e., age, gender, education level, race, and geography), and tobacco user status (current, former, or never) to predict subsequent self-reported purchase. The model includes main effects and interactions to account for different patterns of usage based on demographics variables and tobacco user status, resulting in different predictive relationships between ratings and actual behavior for subgroups defined by the prediction variables. By accounting for differences in the way the scale is used by population subgroups, the algorithm projects more accurate estimates of future purchase than simply using a single uniform algorithm for all respondents. In other words, a given rated intent to purchase may translate into different actual use rates depending on whether the rating was made by a former or never user of tobacco, for example. The product of the analysis was a set of equations using the rated intent, age, and tobacco user status.

The following table lists the model coefficients for demographic groups that were significant predictors of purchase. The final model/algorithm included tobacco use status and the interaction of tobacco use status and age:

**Logistic Regression Results for Key Subgroups**

| ***Model Inputs:*** | Parameter/Coefficient | Standard Error |
| --- | --- | --- |
| Intercept | -6.4986 | 0.3319 |
| Raw Rating Score (1-10) | 0.3367 | 0.0315 |
| Current Regular Tobacco User | 1.7425 | 0.3509 |
| Current Regular Tobacco User  Age 18-30 | 0.843 | 0.2718 |
| Current Regular Tobacco User  Age 31-50 | 0.5153 | 0.228 |
| Former Regular Tobacco User  Age 18-30 | 1.6292 | 0.5775 |
| Former Regular Tobacco User  Age 31-50 | 1.9444 | 0.4161 |

The net result is a highly predictive model with a C-statistic (also known as the area under the receiver operating characteristic curve, a goodness of fit measure that captures the probability of correctly classifying an individual’s actual purchase or non-purchase based on the predictors) of 0.89.

A further study, in which 13,178 consumers participated in the initial wave and 6,469 returned to participate in the second (follow-up) wave, was conducted with a different tobacco product to validate the algorithm. A model was again built for the data from the validation study using the same procedures as used for the original model. No new variables contributed significantly to predicting purchase behavior, and a series of t-tests that compared the coefficients for the second study did not differ significantly from those of the first. However, the intercepts were different. Related to this, the model consistently over-predicted purchase overall and among each of the tobacco user groups. This suggests that the absolute purchase rate may have been influenced by external market factors (e.g., less distribution or promotion), but that the coefficients predicting likelihood of purchase from intent ratings were replicated.

The algorithm was developed and validated using a new-to-market cigarette but has also been used to predict purchase of multiple smokeless tobacco products (including Camel Snus) with similar results.

**Advertisement with Modified-risk Information (test condition)**

[All four government-mandated warning labels tested, with identical advertising content]

**Advertisement without Modified-risk Information (control condition)**

[All four government-mandated warning labels tested, with identical advertising content]
